# Understanding driver behaviour after consuming conventional wines with different sulphite contents: Insights from a driving simulator study

**DOI:** 10.1371/journal.pone.0345134

**Published:** 2026-07-22

**Authors:** Marco Bassani, Camilla De Paolis, Vincenzo Gerbi, Simone Giacosa, Abrar Hazoor, Alessandra Lioi, Maria Alessandra Paissoni, Alberto Portera, Andrea Renolfi, Susana Río Segade, Luca Rolle, Luca Tefa

**Affiliations:** 1 Department of Environment, Land and Infrastructure Engineering, Politecnico di Torino, Torino, Italy; 2 Department of Agricultural, Forest and Food Sciences, Università degli Studi di Torino, Torino, Italy; 3 Business, School, Nord University, Stjørdal, Norway; University of Catania, ITALY

## Abstract

**Background:**

Alcohol consumption is a contributing factor to traffic crashes and road fatalities worldwide. While the effects of blood alcohol concentration (BAC) on driving performance are well documented, less is known about the role of sulphites, used for their antimicrobial and antioxidant properties, on driving performance.

**Method:**

Using a triple-blind, randomised, within-subjects design, thirty-two participants completed three driving simulation trials under sober conditions and after consuming a conventional red wine with ‘low’ (86 mg/l) and ‘high’ (126 mg/l) sulphite content, while maintaining a BAC at about the Italian legal limit (0.5‰). The driving behaviour was assessed in rural, urban and rural-urban transition environments

**Results:**

In less demanding rural environments, where participants drove in free-flow conditions (i.e., rural and transitional), only BAC affected lateral vehicle control along curves, increasing the standard deviation of lateral position relative to the sober baseline, but no significant differences emerged between the two sulphite levels. However, in the more demanding urban environment, when drivers consumed wine with higher sulphite content, they showed more cautious behaviour during sudden interactions with pedestrians at zebra crossings compared with the sober condition. Conversely, in a car following task, when they drank high-sulphite wine, they behaved more dangerously, maintaining higher speeds and shorter headways with respect to the baseline.

**Conclusions:**

Within legal BAC limits, higher sulphite content in wine was associated with context-dependent changes in driving behaviour, promoting greater caution in complex urban scenarios but riskier behaviour in car-following tasks. The small number of significant effects supports the effectiveness of current legal limits, and the findings should be interpreted strictly within regulated conditions, while highlighting the need for further research on sulphite-related effects.

## Introduction

Driving under the influence of alcohol is the cause of a high number of traffic crashes and fatalities. The National Highway Traffic Safety Administration [1] found that in 32% of fatal crashes in 2022, at least one of the drivers involved had a blood alcohol concentration (BAC) greater than 0.08 g/dl, the legal limit for driving a passenger car in the US. Based on official data from 30 European countries, 25% of all road deaths in 2021 were caused by alcohol abuse [2]. In Italy, the percentage of drivers who tested positive for alcohol in 2022 was 10.8%, regardless of whether a road crash occurred [3]. As the BAC increases, the risk of being involved in a fatal accident increases exponentially. Drivers with a BAC between 0.1 and 0.5 g/l are one to three times more likely to be involved in a fatal collision than sober drivers, while the risk increases to five to ten times higher with a BAC between 0.5 and 0.8 g/l [2].

Alcohol affects the regions of the brain responsible for decision-making, inhibiting risky behaviour, and processing sensory information crucial to driving, such as vision and hearing. Research has shown that as BAC increases, driving becomes more aggressive with an increase in average speed [4–6], lateral control of the vehicle is impaired [5,7], reaction time to external events increases, and the safety distance maintained while driving decreases [7]. The extent of these behaviours varies with driving experience, the amount of information to be processed and alcohol consumption.

While the effects of alcohol on driving performance have been the focus of many studies, there is a lack of research on the effects of specific characteristics of alcoholic beverages, particularly when raw materials and production methods introduce variation between beverages that are typically considered similar, such as wine. Many of the chemical characteristics responsible for the diversity of wines are strictly dependent on the grape variety and their winemaking strategy, including the oenological adjuvants used that permit to preserve the quality and longevity of the wine [8].

Sulphur dioxide (SO_2_) is the most widely used preservative additive in winemaking and conservation due to its antimicrobial and antioxidant properties [9]. In oenology, the terms sulphur dioxide, SO_2_, and sulphites are generally used synonymously. In Europe, the limit for conventional wines is set at 150 mg/l of total SO_2_ for red wines and 200 mg/l for white wines if the sugar content is lower than 5 g/l [10], but the limit may be different in other non-European countries. For the European Union and other legislation, producers must indicate on the label the phrase “contains sulphites” if the content is higher than 10 mg/l.

Most strains of *Saccharomyces* yeasts produce modest amounts of sulphites [11]. Therefore, producing totally sulphite-free wines is difficult. However, today, a better understanding of the mechanisms of action of sulphur dioxide has enabled its limited use in commercial wines, alongside appropriate technological choices.

Possible allergenic effects of sulphites in sensitive individuals have prompted producers and researchers to rationalise the use of SO_2_. Gao et al. [12] and Vally and Thompson [13] pointed out that excessive SO_2_ has negative effects on human health with symptoms such as headaches, nausea and asthmatic reactions. These effects may influence the behaviour and performance of those who drive after drinking a legal quantity of wine.

While there is extensive research linking alcohol consumption to impaired driving, to the best of the author’s knowledge, no study has examined the influence of sulphites in complex alcoholic beverages on driving performance.

This study aims to analyse the effect of sulphite content in conventional wine on driving performance. As part of the VINALIA project supported by Fondazione CRC (Cuneo, Italy), two red wines with different sulphite contents (i.e., low, 80 mg/l; high, 120 mg/l) were prepared, below the legal SO_2_ limit for ‘conventional wines.’ A driving simulation experiment was then designed with participants who drove under (i) sober conditions (baseline), and after drinking the wine with (ii) low and (iii) high sulphite content. It is specified that the administration of wine to the volunteers participating in the driving experiment was intended to maintain their BAC at 0.5‰, i.e., the legal limit for driving in Italy under art. 186 of the Italian Highway Code [14].

## Materials and methods

The DISAFA (Department of Agricultural, Forest and Food Sciences, *Università degli Studi di Torino*) produced the wines in its experimental winery. The bottles of wine were then sent to the Road Safety Laboratory of DIATI (Department of Environment, Land and Infrastructure Engineering, *Politecnico di Torino*), where they were administered to the participants to assess the effect of sulphite content on driving behaviour.

### Wine production

About 200 kg of red Barbera grapes (*Vitis vinifera L.*) were destemmed and crushed using an industrial crusher-destemmer (Enoveneta, Piazzola sul Brenta, Italy). The must and grape solid parts were placed into CO_2_ saturated stainless-steel tanks and inoculated for the alcoholic fermentation with 20 g/hl of *Saccharomyces cerevisiae* active dry yeasts (Lalvin BRL 97, Lallemand Inc.). During the alcoholic fermentation- maceration, the temperature (26 ± 1 °C) and the sugar reduction were monitored daily. Two additions of 15 g/hl of nutrients (Fermaid E, Lallemand Inc.) were made. Moreover, two punch-downs per day were carried out in the first three days, followed by two pumping-overs until the end of maceration, which lasted eight days. Later, the pomace cap was pressed (PMA4, Velo SpA, Altivole, Italy), and the pressed wine was added to the free-run part. Then, 1 g/hl of lactic bacteria *Oenococcus oeni* VP41 MBR ML (Lallemand Inc.) were added to induce the malolactic fermentation. At the end of malolactic fermentation, the wine was racked and ~50 mg/l of SO_2_ added by use of potassium metabisulphite (E224). The wine was then stored at 0 °C for two weeks for cold stabilisation and finally filtered and bottled. SO_2_ was added directly to the bottles to achieve the desired final level. For the ‘high’ total SO_2_ content (~120 mg/l), ~ 70 mg/l of SO_2_ was added. Instead, for the samples with ‘low’ amounts of total SO_2_ (~80 mg/l), ~ 30 mg/l of SO_2_ were added to the bottles using potassium metabisulphite. The bottled wines were used for driving simulation experiment tests after three months of storage, starting from the addition of potassium metabisulphite, to reduce the ‘free’ fraction of SO_2_, which is more sensorially perceivable compared to ‘bound’ SO_2_ [8].

As for the basic parameters of the wines produced, the total sugar content was less than 1.0 g/l and the ethanol content (% v/v) was 15.2. The total acidity was 6.31 g/l expressed as tartaric acid, and the pH was 3.50. The main organic acids were: 0.43 g/l acetic acid, 1.64 g/l tartaric acid and 1.58 g/l lactic acid. Malic acid was not detected, indicating that malolactic fermentation was complete. As far as the total SO_2_ content is concerned, the bottled wines with the higher content reached values of 126 mg/l. In the bottled wines with the low level, 86 mg/l of total SO_2_ were detected. Concerning the phenolic composition, the content of total anthocyanins was 324 mg/l as malvidin-3-O-glucoside chloride, while the content of total flavonoids was 1248 mg/l as (+)-catechin. The colour intensity and hue of the wine were determined, and the results were 9.17 absorbance units and 0.81, respectively.

### Driving simulation experiment

#### Experimental design.

This study used a triple-blind, randomised, within-subject experimental design. During the driving simulation experiments and data analysis at DIATI, only the DISAFA researchers knew the characteristics of the wines. All drivers completed trials under all conditions, resulting in multiple measurements per participant (repeated measures). Each participant was involved in three driving sessions on different days under (i) sober conditions (baseline), (ii) after drinking 1.5 units of alcohol in the wine with low SO_2_ content (86 mg/l), and (iii) after drinking 1.5 units of alcohol in the wine with high SO_2_ content (126 mg/l). The order of the driving sessions was randomised. One unit of alcohol contains 12 grams of pure alcohol, e.g., about 300 ml of beer or 100 ml of red wine [15]. The value of 1.5 units of alcohol was chosen to give a BAC peak of 0.5‰, which is around the legal limit for driving in Italy (i.e., 0.05 g/dl). Given that the Alcohol by Volume (ABV) of the wine was 15.20%, 190 ml of wine was administered.

#### Driving simulator.

The fixed-base driving simulator of DIATI was used, consisting of a cockpit with a force‑feedback steering wheel, manual gearbox, pedals and a dashboard with a speedometer and rev counter displayed on a small monitor mounted behind the steering wheel. A three‑screen vision system with a resolution of 1920 × 1080 pixels, a refresh rate of 60 Hz, and a field of view of 130◦ (H) × 20◦ (V) was provided. The driving simulator was behaviourally validated for longitudinal [16], lateral [17] and passing behaviour [18], as well as for tunnel environments [19], ensuring its reliability as an effective research tool.

#### Participants.

All participants in the experiment responded voluntarily to an invitation sent via social communication channels (Facebook, LinkedIn) and by email to contacts from previous experiments. It was explained that participation was voluntary and without financial compensation. To limit the variance in the sample due to personal alcohol tolerance, total abstainers and habitual drinkers, i.e., those consuming more than four units of alcohol per week, were excluded. Only individuals of Caucasian ethnicity who consumed less than fifteen cigarettes and five coffees per day were included to avoid any possible correlation with tobacco and caffeine consumption. Self-reports were used to exclude those who used drugs, had psychiatric disorders, head injuries or other central nervous system disorders, or were pregnant. No minors were involved; only drivers with a European B (or higher) driving licence, which permits the holder to drive standard passenger cars (higher licence classes allow for additional or larger vehicle types). The recruitment period began on 8 July 2022 and concluded on 29 November 2022. Out of the 183 individuals who responded to the invitation, 82 met the selection criteria for the experiment. From these, 32 drivers were randomly selected, ensuring equal numbers of men and women (see Table 1). The sample size of 32 participants was justified by an a priori G*Power analysis for the within-subject repeated-measures design, which indicated a statistical power of .95

**Table 1:** Mean and standard deviation (in brackets) of drivers’ demographic characteristics.

Gender	No.	Age, years	Experience, years	Experience, km/years	No. of crashes
Male	16	33.4 (9.2)	15.0 (9.3)	7 687.5 (7 162.1)	1.4 (1.4)
Female	16	34.3 (11.6)	15.5 (11.8)	5 518.8 (3 638.5)	0.9 (0.8)
Total	32	33.9 (10.3)	15.3 (10.4)	6 603.1 (5 695.6)	1.1 (1.1)

In accordance with the Ethical Committee’s guidelines (see Section 2.2.8), an informed consent form was sent to participants via email before the experiment, and written consent was obtained before the driving simulation. Participants gave written permission for their information to be used.

#### Protocol.

Participants were asked to adhere to the following protocol: (i) not to consume any alcoholic substances the day before the test; (ii) to sleep for at least seven hours the night before the test; (iii) not to eat breakfast the morning before the test or (if this was not possible for medical reasons, to eat a light, fat-free breakfast); (iv) not to consume any other alcoholic substances on the day of the experiment; (v) not to drive within one hour after the test.

The appointments were scheduled between 7.30 am and 10 am to allow the drivers to take part in the tests on an empty stomach, which favours the appearance of the BAC peak after 20 min [20]. Each meeting was scheduled at least one week apart to minimise any learning effect.

On arrival at the laboratory, the driver’s BAC was measured to confirm that the subject had not been drinking before the test and to calibrate the breathalyser, i.e., to ensure that it was working properly. Before starting the experiment, the drivers drove on a test track for five minutes to familiarise themselves with the driving simulator and to check that the experience did not cause any symptoms of simulation sickness. None of the test drivers reported any such symptoms. After the pre-test drive, the subject was given the wine at two randomly selected appointments out of three. Twenty minutes after drinking, the BAC was measured again, and then the participant performed the experimental session, which lasted approximately ten minutes. Finally, the final BAC (*BAC*_*F*_) was measured for the last time. At the end of the experiment, the subject was offered breakfast to help metabolise the alcohol intake and minimise any influence for the rest of the day.

#### Road scenarios.

**Fig 1:**
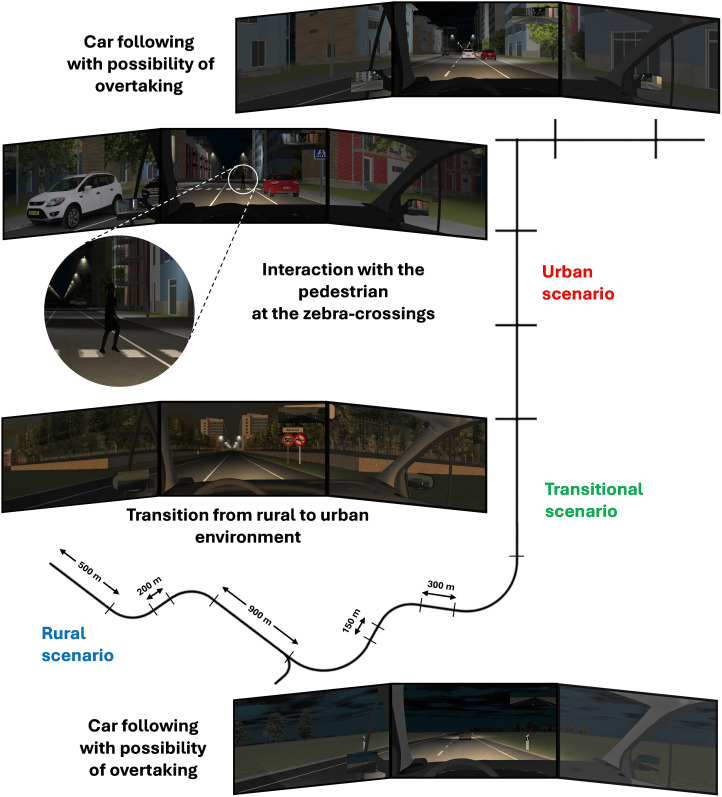
Plan scheme of the road network, with the vision from the driver’s point of view of the rural, transitional and urban road scenarios.

The experimental track was designed in accordance with Italian technical standards [21]. It started with 5 km of two-lane rural road (road type C1, speed limit 90 km/h, two 3.75 m wide lanes, paved shoulders 1.5 m wide), consisting of a series of tangents and curves of large radii (higher than 300 m) connected by spirals (Fig 1). On one of the straight sections, the driver could overtake a slow car travelling at 60 km/h. Vehicles travelling in the opposite direction were excluded to facilitate overtaking.

After a transition from rural to urban (Fig 1), the participants drove 4 km in an urban scenario with a speed limit of 50 km/h (road type E, two 3 m wide lanes, 2.2 m parallel parking lanes on both sides, and 2.5 m wide sidewalks). It was designed with two 2-km long straight sections joined at a signalised intersection. In the first section, participants occasionally interacted with pedestrians crossing randomly from both sides of the zebra crossings to avoid any learning effect. In the second section, a car-following scenario was simulated in which a vehicle travelling at 45 km/h entered the road ahead of the ego-vehicle. Night conditions were simulated in all driving sessions (Fig 1).

#### Dependent variables.

In the rural environment, longitudinal and lateral control were assessed using various speed and lateral position indicators, respectively. The mean (*tangent_S*) and the standard deviation of speeds (*tangent_SDS*) along the straight segments, the mean speed at the midpoint of the curves (*curve_S*), and the standard deviation of lateral position along the tangents (*tangent_SDLP*) and curves (*curve_SDLP*) were considered when evaluating the effects of the different treatments. If speed is the primary indicator of driving ability and risk [22], the standard deviations of speed and lateral position within the lane indicate the ability to maintain longitudinal and lateral control of the vehicle [23,24].

In the rural-urban transition section, several indicators of time, distance and speed were estimated to understand how drivers adapted their behaviour to the new driving conditions. Among these indicators, only the transition time (*trans_time*), i.e., the time required to adjust the vehicle speed to the urban speed limit, was sensitive to the experimental treatment. The shorter the *trans_time*, the faster the adaptation to the new driving conditions.

In the urban environment, we simulated two different driving events. In the first, we investigated the response to a sudden interaction with a pedestrian who was suddenly visible a few seconds before the ego vehicle reached the zebra crossing. We adopted a pedestrian time gap acceptance of 5 s, which Angioi and Bassani [25] found to be critical for safety when pedestrian interacts with sober drivers. To increase the event’s suddenness, the pedestrian’s movement during the crosswalk occupancy phase was obscured by vehicles parked along the parking lane, as shown in Fig 1. In the second, participants had to interact for approximately 800 m with a vehicle in the same lane travelling at 45 km/h, a speed lower than the 50 km/h limit. That vehicle merged safely into the lane without the participants having to adjust their speed.

Proximity measures of safety, such as the Minimum Time-to-Collision (*MTTC*) and the Post-Encroachment Time (*PET*), were used to evaluate the safety of vehicle-pedestrian interactions [26,27]. The *MTTC* measures the minimum time that separates two road users from a collision if they do not change their direction and/or speed. The *PET* measures the temporal distance between road users when one leaves the conflict area and the other enters it.

Both *MTTC* and *PET* were evaluated for conflicts with pedestrians crossing from the right side only. A preliminary analysis showed that the interaction with the pedestrian crossing from the left did not yield statistically significant results because drivers had more time to react by stopping before the crosswalk, even after consuming wine.

For the car-following scenario simulated in the second urban section, the mean (*urban_S*) and maximum speed (*urban_maxS*), the lateral position from the lane centreline (*urban_LP*), as well as the standard deviations of speed (*urban_SDS*) and lateral position (*urban_SDLP*), were considered. Additionally, we evaluated the mean (*urban_HW*), minimum (*urban_minHW*), and standard deviation (*urban_SDHW*) of headway time relative to the lead vehicle.

The driver actions on longitudinal and lateral control systems in such a monotonous condition could be altered by the alcohol consumption as observed in Berthelon and Gineyt [28].

#### Independent variables and data modelling.

Linear mixed-effects models (LMMs) were used to assess the effects of the independent variables on the selected behavioural variables. LMMs allow fixed (common factors) and random effects (group or subject-specific variability) to be modelled simultaneously, providing a more realistic representation of the data. Using LMMs can yield more accurate estimates and more reliable inferences, reducing the risk of Type I errors and improving statistical power. For the random effects in the model, *Test_driver_ID* was considered as a cluster variable. For the fixed effects, (i) *Gender* and (ii) *Sulphite_content* of wines were considered as categorical factors, while (iii) *Age*, (iv) *BAC*_*F*_ at the end of the driving session, the *Driving_experience* in terms of (v) *years* of driving licence, and (vi) average driven in a year (*km/y*) were included as covariates.

A stepwise model selection process was used to optimise LMM performance, along with backward elimination to systematically remove statistically non-significant variables and interactions. Model fit was assessed using the Akaike Information Criterion (AIC) [29] and the Bayesian Information Criterion (BIC) [30] to improve model quality. For variables and interactions that were statistically significant, post hoc comparisons were performed using the Holm correction for multiple comparisons. The significance level was set at .05.

LMM were estimated using the *lme4* package [31] of R statistical software version 4.3.2 [32]. The *emmeans* package [33] was used to calculate the estimated marginal means. Plots of the results were generated using the *ggplot2* package [34].

### Ethics approval

Experimental design and data collection in the present study have been approved by the Ethics Committee of the Politecnico di Torino (Decision No. 15681, 8 April 2022).

## Results

A summary of the results of the effects of the wine treatment, together with the characteristics of the participants (age, weight, height and body mass index), is given in Table 2. The BAC of the untreated case that was taken before the start of the driving tests is also included. Of the three BAC measurements taken at each appointment (see Section 2.2.4), only the final value (*BAC*_*F*_) taken immediately after the end of the driving is included in Table 2. It is worth noting that *BAC*_*F*_ measured in females was significantly higher than that in males due to the lower body mass index and the different metabolic kinetics between the two genders [35,36].

**Table 2:** Mean and standard deviation (in brackets) of BAC and drivers’ characteristics. (Note: BMI = Body Mass Index; BMI is calculated by dividing weight in kg by height in metres squared).

Gender	BAC_F_, ‰			Age years	Weight kg	Height m	BMI kg/m^2^
	SO_2_ = 0 mg/l	SO_2_ = 86 mg/l	SO_2_ = 126 mg/l				
Female	0.000 (0.000)	0.540 (0.167)	0.550 (0.182)	34.3 (11.6)	68.3 (22.2)	1.65 (0.062)	24.9 (7.6)
Male	0.000 (0.000)	0.398 (0.114)	0.402 (0.153)	33.4 (9.2)	80.7 (12.9)	1.78 (0.070)	25.5 (3.7)
Total	0.000 (0.000)	0.469 (0.158)	0.476 (0.182)	33.9 (10.3)	74.5 (18.9)	1.710 (0.091)	25.2 (5.8)

### Rural environment

Table 3 provides the synthesis of the results obtained in the rural environment, with data representing the longitudinal (i.e., mean and the standard deviation of speeds along the straight segments and curves midpoint) and the lateral (i.e., mean and standard deviation of lateral position along tangents and curves) behaviour of drivers sober (baseline) and after the treatments (intake of wine under different sulphite content).

**Table 3:** Descriptive statistics of driving performance measures in the urban environment, both at baseline (no alcohol intake) and after wine consumption with different sulphite contents.

Variables	u.m.	Baseline	86 mg/l	126 mg/l
*tangent_S*	km/h	85.7 (9.76)	84.5 (8.64)	86.1 (8.25)
*tangent_SDS*	km/h	0.905 (0.507)	0.846 (0.632)	0.858 (0.485)
*tangent_SDLP*	m	0.192 (0.085)	0.202 (0.0992)	0.186 (0.0998)
*curve_S*	km/h	83 (9.86)	82.1 (9.52)	83.1 (8.45)
*curve_LP*	m	−0.0877 (0.195)	−0.114 (0.2)	−0.0738 (0.202)
*curve_SDLP*	m	0.205 (0.0556)	0.23 (0.0672)	0.224 (0.0673)

Table 4 shows the LMM results for the driver behaviour on tangents and curves. Here, there was no evidence of effects attributable to the sulphite treatment through the intake of the wine, while in only one case, *BAC*_*F*_ was found to have a significant effect on driver behaviour, as follows.

**Table 4:** LMM results of the rural environment for tangents and curves (Note: * for p < .05, ** for p < .01, and *** for p < .001, symbol – means not statistically significant; LRT = likelihood ratio test).

Variables	Estimates			
	*tangent_SDS,* km/h	*tangent_SDLP*, m	*curve_S,* km/h	*curve_SDLP,* m
**Fixed effects**				
*Intercept*	0.226 (-)	0.095 (-)	82.773 (***)	0.205 (***)
*Age*	0.019 (*)	0.003 (*)	–	–
*BAC*_*F*_	–	–	–	0.046 (**)
**Random effects**				
*Test_driver_ID* (LRT)	(***)	(***)	(***)	(***)
**Summary statistics**				
AIC	146.542	−196.144	666.205	−280.899
BIC	156.800	−185.887	673.898	−270.642
Log – Likelihood	−69.271	102.072	−330.102	144.450
Marginal R^2^	.126	.096	.000	.036
Conditional R^2^	.485	.632	.621	.654
ICC	.411	.592	.621	.641

Along straight sections, *Age* had a significant effect (*p* = .010) on the standard deviation of speeds (*tangent_SDS*), suggesting that, among older drivers, longitudinal control deteriorates (i.e., *SDS* increases). The random effect was highly significant, indicating a relevant influence of driver subjectivity. The variance explained by the fixed effects alone was relatively small (marginal R² = .126), while about 40% of the variance was attributable to differences in driver ability, underscoring the importance of this cluster variable in the model.

Lateral control on straight sections (*tangent_SDLP*) also deteriorated significantly with age (*p* = .040). Like longitudinal control, the random effect was highly significant, with an ICC of .592, indicating a strong individual influence on the variance in the data. With 63.2% of the total variance explained, the model performed well overall.

For the speed at the midpoint of the curves (*curve_S*), only the intercept was significant (*p* < .001), indicating that participants adjusted speed according to their personal judgement, with no experimental factors influencing their choices. In fact, the random effect of drivers was again highly significant, with an ICC of 0.621, highlighting substantial intersubject variability.

Finally, *BAC*_*F*_ was the only significant factor (*p* = .004) for the standard deviation of lateral position along curves (*curve_SDLP*), suggesting that as *BAC*_*F*_ increases, the lateral control deteriorates. Again, the random effect of subject was highly significant (ICC = .641). The variance explained by *BAC*_*F*_ was of 3.6% of the total.

### Transition zone

In the transition zone, the speed limit was changed from 90 (rural) to 50 km/h (urban). Table 5 presents a summary of the results showing the time and length required for drivers to adapt their longitudinal behaviour both when sober (baseline) and after consuming wine with varying sulphite content. Also, the maximum and standard deviation of speed are provided.

**Table 5:** Descriptive statistics of driving performance measures in the transitional zone, both at baseline (no alcohol intake) and after wine consumption with different sulphite contents.

Variables	u.m.	Baseline	86 mg/l	126 mg/l
*trans_time*	s	19.8 (6.49)	16.1 (7.79)	17.3 (8.69)
*trans_length*	m	369 (121)	304 (148)	332 (179)
*trans_Smax*	km/h	87 (8.61)	86.7 (9.36)	87.9 (7.95)
*trans_SDS*	km/h	12 (3.04)	11.8 (3.14)	11.5 (2.58)

In LMM, random effects were included to account for driver-related variability, which was previously found to be significant in the rural road section. Among the four variables, we found that only the duration of the transition time (*trans_time*) to adapt the speed was statistically influenced by *Gender* (*p* = .031), with male drivers taking, on average, 4.1 s less to complete the speed variation than females. The *Test_driver_ID* was highly significant, indicating considerable variability among subjects. The fixed effects explained a limited amount of variance in the model (marginal *R*² = .070), while only 24.8% of the total variance was explained by fixed and random effects.

### Urban environment

In contrast to the previous cases, different effects on driving performance and safety measures (Table 6) were observed in the urban context due to the different sulphite treatment and blood alcohol content resulting from wine consumption. The results of the two main urban sections are presented in the following subsections.

**Table 6:** Descriptive statistics of driving performance measures in the urban environment, both at baseline (no alcohol intake) and after wine consumption with different sulphite contents.

Variables	u.m.	Baseline	86 mg/l	126 mg/l
*MTTC*	s	3.53 (1.98)	2.92 (1.34)	3.4 (3.19)
*PET*	s	5.72 (2.51)	5.04 (1.92)	5.08 (2.05)
*urban_S*	km/h	47 (2.54)	46.9 (2.09)	47.6 (2.42)
*urban_maxS*	km/h	52.9 (4.6)	53.6 (4.86)	54.3 (4.77)
*urban_SDS*	km/h	2.77 (1.34)	2.96 (1.5)	3.31 (1.41)
*urban_LP*	m	0.0697 (0.19)	0.0192 (0.182)	0.0169 (0.151)
*urban_SDLP*	m	0.142 (0.0343)	0.16 (0.054)	0.15 (0.0531)
*urban_HW*	s	6.84 (4.06)	5.73 (3.8)	5.7 (3.12)
*urban_SDHW*	s	1.27 (0.837)	1.09 (0.453)	1.27 (0.718)
*urban_minHW*	s	4.81 (3.43)	4.07 (3.44)	3.74 (2.77)

#### First section with pedestrians’ interaction.

In the first urban section, participants interacted with pedestrians at zebra crossings. Table 7 summarises the results of the LMMs calibrated for the *MTTC* and the *PET*, the main safety indicators used to understand the vehicle-pedestrian interaction. The higher the *MTTC* and *PET* values, the safer the interactions between the driven vehicles and the simulated pedestrians.

**Table 7:** LMM results of the pedestrian interaction in the urban environment (Note: * for p < .05, ** for p < .01, and *** for p < .001, symbol – means not statistically significant; LRT = likelihood ratio test).

Variables	Effect	Estimates	
		*MTTC*, s	*PET*, s
**Fixed effects**			
*Intercept*		5.010 (***)	5.751 (***)
*Sulphite_content*	0 – low	1.962	–
	0 – high	2.485 (*)	–
*BAC*_*F*_		−5.500 (**)	−1.494 (*)
*Driving_experience* (km/y)		0.066 (*)	–
*Driving_experience* (y)		−0.098 (*)	–
**Random effects**			
*Test_driver_ID* (LRT)		(-)	(***)
**Summary statistics**			
AIC		433.385	412.146
BIC		453.900	422.403
Log – Likelihood		−208.692	−202.073
Marginal R^2^		.175	.032
Conditional R^2^		.281	.423
ICC		.129	.404

Compared to the baseline (*BAC*_*F*_ = 0, *Sulphite_content* = 0), a low sulphite content had a marginally significant effect on *MTTC* (*p* = .051), increasing *MTTC* by about 2 s, while the higher SO_2_ content produced a statistically significant increase of about 2.5 s (*p* = .015). *BAC*_*F*_ had a statistically significant effect on *MTTC,* but it worked in a different way with respect to the sulphite content of the wine, i.e., the higher the BAC, the lower the *MTTC*. Driver experience was also found to be significant in the model. If the number of km driven in a year increased *MTTC* (*p* = .011) thus improving safety, a higher driving age significantly decreased *MTTC* (*p* = .044). The random effect was not significant (*p* = .241), and the fixed effects accounted for only 17.5% of the total variance in the model.

On *PET*, *BAC*_*F*_ had a significant effect (*p* = .034), confirming that alcohol reduces safety by increasing the risk of dangerous interactions when the pedestrian has left the conflict area. However, there was no significant effect of sulphites on either *PET* or driving experience. In contrast to *MTTC*, the random effects were highly significant. In support of this, the fixed predictors explained only 3.2% of the variance, while the individual characteristics and explained most of the variance in the model (ICC = .404).

#### Second section with car-following scenario.

For the car-following scenario experienced by participants on the second urban road section, Table 8 summarises the LMM results, showing distinct effects across factors.

**Table 8:** LMM results of the car following in the urban environment (Note: * for p < .05, ** for p < .01, and *** for p < .001, symbol – means not statistically significant; LRT = likelihood ratio test).

Variables	Effect	Estimates (p-value)			
		*urban_maxS*, km/h	*urban_SDS*, km/h	*urban_SDLP*, m	*urban_minHW*, s
**Fixed effects**					
*Intercept*		51.930 (***)	1.226 (-)	0.138 (***)	4.912 (***)
*Sulphite_content*	0 – low	0.159 (-)	0.189 (-)	–	0.108 (-)
	0 – high	2.814 (**)	0.545 (*)	–	−1.483 (*)
*Gender*	M – F	1.884 (-)	–	–	−0.208 (-)
*Sulphite_content* * Gender	(0 – low) * (M – F)	1.092 (-)	–	–	−1.696
	(0 – high) * (M – F)	−2.816	–	–	0.832 (-)
*BAC*_*F*_		–	–	0.040 (**)	–
*Age*		–	0.045 (*)	–	–
**Random effects**					
*Test_driver_ID* (LRT)		(***)	(***)	(***)	(***)
**Summary statistics**					
AIC		535.400	314.888	−315.929	466.459
BIC		555.915	330.274	−305.671	486.974
Log – Likelihood		−259.700	−151.444	161.964	−225.230
Marginal R^2^		.062	.128	.049	.050
Conditional R^2^		.623	.653	.505	.627
ICC		.599	.602	.479	.608

Regarding the maximum speed of drivers in the section (*urban_maxS*), the higher sulphite treatment significantly increase in speed (*p* = .010). *Gender* was not significant as a factor per se, but it had a marginal impact in interaction with sulphites (*p* = .063). Post-hoc tests with Holm correction showed that, contrary to what was found for males, high sulphite content increased the maximum speed of females (*t*_60_ = 2.674, *p*_Holm_ < .087). The model shows a high effect of the individual component (ICC = .599), with a marginal R² of 6.2% and a conditional R² of 62.3%, indicating a relevant subjective influence of drivers.

High levels of sulphites significantly increase speed variability (*urban_SDS*) in car following (*p* = .013), indicating greater instability in participants’ speed control. There is also a significant increase in speed variability with *Age* (*p* = .031), with a highly statistically significant effect of subjective ability and attitude, as evidenced by the high ICC value (.602).

For the lateral control in urban driving (*urban_SDLP*), *BAC*_*F*_ had a relevant effect (*p* = .006), indicating that alcohol intake reduces lateral vehicle control. The model had a marginal R² of only 4.9% but a conditional R² of 50.5%, indicating that almost half of the variance was explained by individual differences between drivers.

Finally, *Sulphite_content* became significant again in the minimum time headway (*urban_minHW*) with the front vehicle travelling at 45 km/h. With the high sulphite content, the minimum headway was significantly reduced compared to the baseline condition (*p* = .042). *Gender* produced a marginally significant interaction (*p* = .098) with the low sulphite content in the wine, as it reduced the minimum headway, especially for males. As with the previous variables, the influence of the subjects’ ability and attitude was also observed for this dependent variable.

## Discussion

In this triple-blind, within-subjects experimental study, we investigated the effects of sulphite content on driving behaviour across three road scenarios: rural, urban, and the transition between them. Data analysis clearly identified the effects of the controlled experimental factors related to the wine treatment (sulphite content and BAC) and the participants’ demographic characteristics.

### Driving performance and safety in the rural context

Our analyses do not show any significant effects of wine consumption in a rural context, because of the driving conditions we simulated, which were mainly free-flowing and low‑demanding. Participants interacted only with a slow car on a very long straight (800 m), and they were able to overtake it easily and safely because no oncoming traffic was simulated. Drinking wine with different levels of sulphite had no effect on the decision to overtake or not to overtake this slow car. The wine only played a role in lateral control along curves, a result consistent with the current literature [37]. Only after drivers consumed the two wines did the standard deviation of lateral lane position increase significantly relative to the baseline (i.e., no wine consumption). The increase in lateral position variability along curves related to BAC matches earlier simulator and test-track findings that alcohol impairs basic lateral control abilities [6,7,24].

It should also be noted that, in this driving context, no driver was at risk of losing control of the vehicle or inadvertently leaving the lane. In summary, no effect was observed due to differences in the sulphite content of the two wines administered.

These results confirm that the BAC limit set by the Italian road traffic regulations (0.5‰) ensures safe conditions when the driver is not required to perform complex tasks [38,39]. In these driving conditions, the observed variability was mainly influenced by participants’ subjective ability and driving style, with only a moderate effect of BAC on vehicle control in curves.

### Driving performance and safety in the urban context

**Fig 2:**
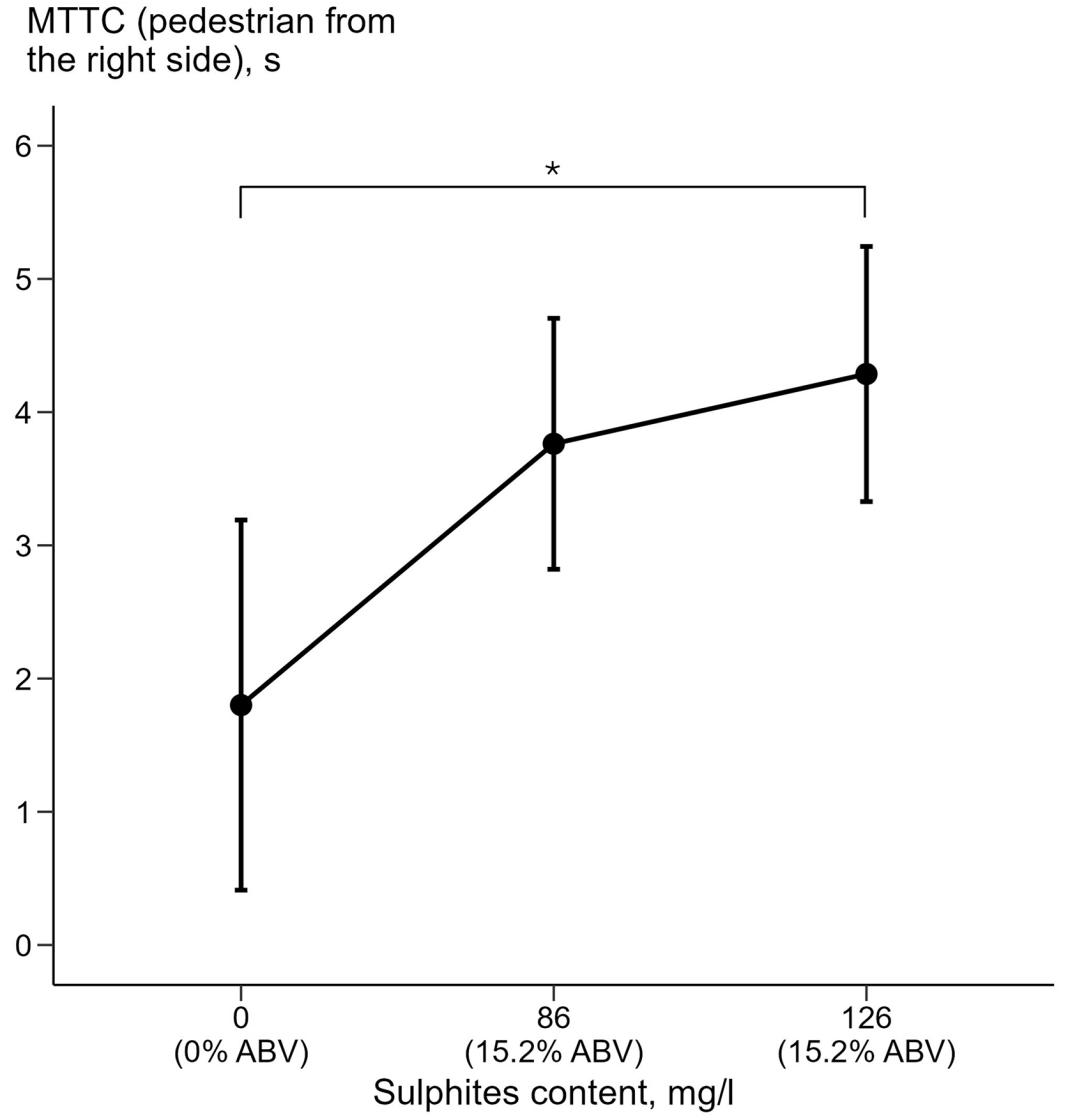
Effect of the sulphite content in the wines on MTTC in the interaction of participants with simulated pedestrians in the first section of the urban environment.

In contrast, driving is more affected by wine intake and the sulphites it contains under more demanding conditions, such as those recreated here in the urban road sections. With regard to the pre-conflict phase (i.e., before the participants interacted with the pedestrians in the zebra crossing) the *MTTC* was significantly increased by the sulphite content in the wine, especially at the high content (Fig 2). This may be a compensatory response to a perceived potential conflict. According to the Task-Capability Interface model, drivers adjust their behaviour—such as speed and safety margins—to keep perceived task difficulty manageable [40]. In this context, the sudden appearance of a pedestrian at the zebra crossing may have heightened perceived task demands, prompting drivers to increase their safety margins. However, caution is warranted in this interpretation, as risk perception and perceived workload were not directly measured in this study. Additionally, the contrasting effects of BAC on MTTC and PET align with prior research indicating that alcohol impairs drivers’ ability to process safety-related information and maintain appropriate safety margins in traffic situations [41].

Experience also played an important role in this driving environment. Subjects with higher annual mileage showed higher *MTTC*, whereas older driver’s licenses were associated with lower *MTTC*. This suggests that not all aspects of experience play the same role in safety, with experience leading to greater skill in control but also less caution in conflicting circumstances [42]. The data reveal no effect of sulphites on the post-event measurement (PET).

**Fig 3:**
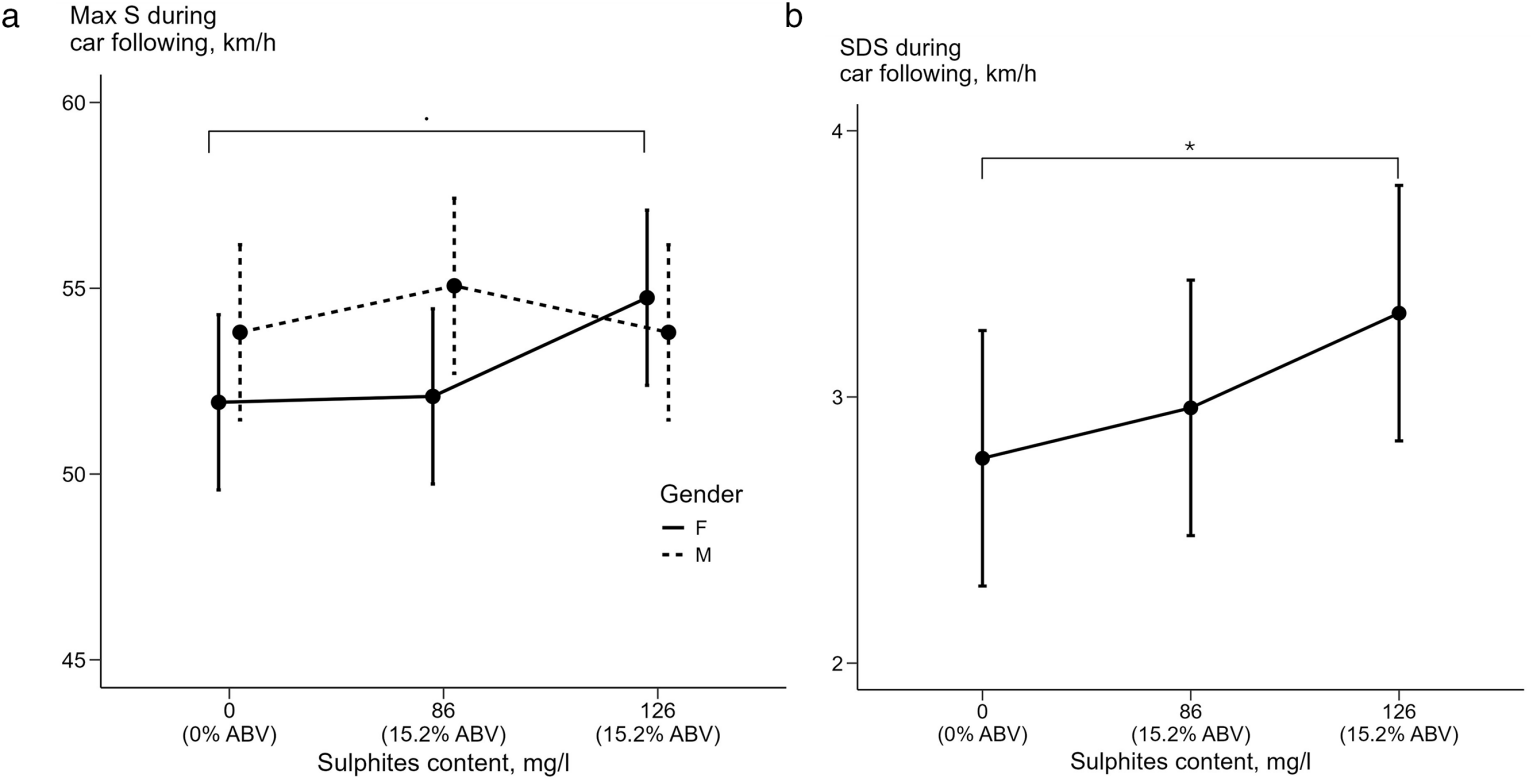
Effect plot for longitudinal behaviour in car following: (*a*) maximum speed and (*b*) standard deviation of speed for different sulphite content in the wines.

The results of the car-following condition in the second section of the urban environment indicate that sulphites and BAC had a slightly negative effect on driving behaviour. It is worth noting that the participants interacted with a slow-moving vehicle in front for about 800 m. Unlike the first urban section, drivers had to maintain the gap with the leading vehicle. We observed that when participants consumed wine with higher sulphite levels, they showed a small but higher maximum speed on urban roads (Fig 3a), a behaviour that reflects a reduced ability to control speed, as clearly shown in Fig 3b. Under the influence of the higher sulphite wine, the drivers showed less time to distance themselves from the leading vehicle, indicating a reduced ability to control the safety margin. Some interactions between sulphites and gender, although of marginal significance, suggest different responses between males and females. The car-following findings suggest a different behavioural mechanism from that observed in the pedestrian-interaction task. Unlike the sudden pedestrian conflict, the car-following task required sustained monitoring of a lead vehicle and continuous regulation of speed and headway. Previous simulator studies have shown that alcohol impairs vehicle control and manoeuvring behaviours, including speed regulation, lane-keeping, and headway measures [7,28]. Ahlström et al. [41] showed that alcohol can impair driver attention and reduce the effectiveness of compensatory strategies. The higher maximum speed, greater speed variability, and shorter minimum headway after high-sulphite wine may indicate that compensatory behaviour did not generalise to prolonged car-following, where maintaining a safe margin depends on continuous attention rather than on a single hazard.

**Fig 4:**
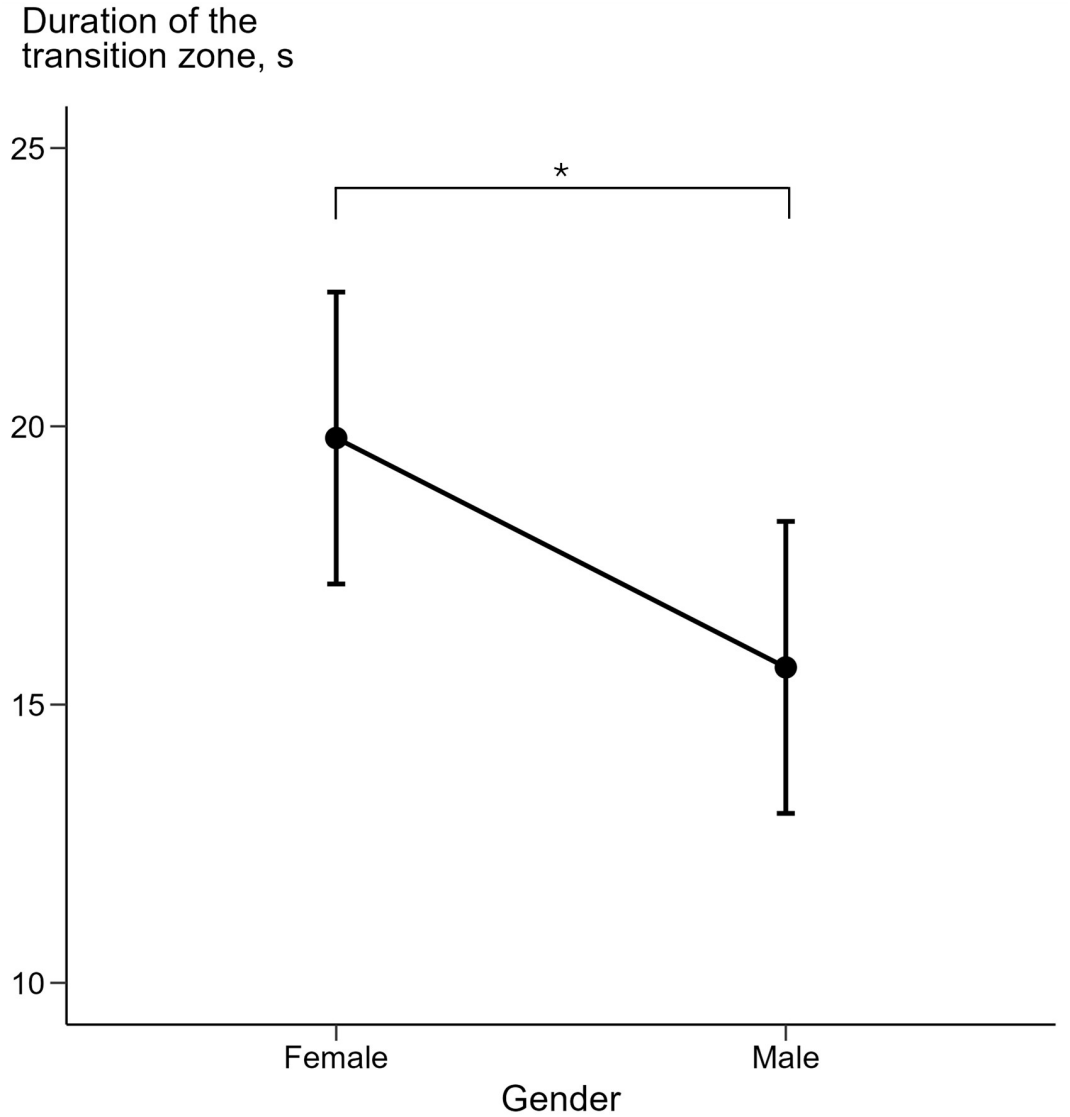
Effect plot of the gender on the duration of the speed variation in the transition zone between rural and urban environments.

Finally, gender influenced the time required to adapt driving speed. Male participants slowed down to the posted speed limit more quickly than female participants (as shown in Fig 4), suggesting possible differences in strategy or response to changes in the road environment. De Craen et al. [43] found that, when observed driving in an urban environment, male drivers adapted their speed better than females. However, there were no gender differences in the station at which the transition manoeuvre began.

### Research implications

This study presents new evidence that, even within legally permitted BAC levels, the sulphite content of wine can influence driving behaviour in a context-dependent way, with effects mainly appearing under high cognitive load and complex interaction demands. By using a triple-blind, within-subjects design and separating sulphite effects from alcohol intoxication, the findings extend current road safety research beyond alcohol consumption alone and highlight the influence of non-alcoholic wine components in shaping driving performance. The results indicate task-specific behavioural adjustments: increased caution in pedestrian‑conflict situations but reduced safety margins during prolonged car-following, suggesting that compensatory behaviour may not apply universally across driving scenarios. Although the limited number of significant effects supports the adequacy of current legal BAC limits, the observed interactions with driving experience and gender indicate notable individual differences.

These results highlight the importance of considering not just BAC but also the sulphite content in alcohol-related driving studies. Earlier research indicated that different wine types could influence behavioural and attentional responses, even when alcohol intake is held constant [4]. Building on this, the current study focused on sulphite levels in traditional wine, demonstrating that their effects depend on context and are primarily observed during complex urban driving tasks. However, because these effects were limited to specific indicators and situations, the findings should be viewed as preliminary, suggesting that non-alcoholic wine components might influence driving behaviour under specific task conditions rather than directly impact driving safety through sulphites.

### Limitations

A limitation of this study is that we did not design the experiment to determine whether, and to what extent, driver impairments (e.g., nausea, dizziness, or oculomotor strain) were caused by the experimental manipulation (wine intake) itself or by simulation-induced discomfort. Although participants did not spontaneously report sickness during the sessions and no trials were stopped because of discomfort, the experiment lacked a systematic assessment of symptoms that might have affected task performance. Future research should incorporate validated protocols and measures of simulation sickness to better control for these potential confounding factors and to improve the interpretation of driving performance and safety data.

### Future needs

Future research should explore a broader range of sulphite concentrations, include physiological and subjective measures, including sickness questionnaires and protocols capable of distinguishing the effects of simulations from those associated with the wine intake, and examine longer, more demanding driving scenarios to better understand the underlying mechanisms and potential implications for road safety under compliant legal conditions.

## Conclusions

The consumption of alcoholic beverages, such as wine, before driving is restricted by law, with varying limits across countries. Many people are sensitive to sulphites, experiencing headaches, nausea and asthmatic reactions, so some producers have rationalised their use in wine.

Taken together, the results of this study show that the consumption of wine with a higher sulphite content influences driving performance in those environments that require greater attention and caution due to riskier interactions with other road users, and that generally require greater longitudinal and lateral control to avoid dangerous interactions with moving vehicles, as well as crossing pedestrians. In this study, these different interactions were manipulated in the urban scenario, whereas in the rural scenario, drivers benefited from interaction-free driving, with the sole exception of a low-speed vehicle, which many participants had no difficulty overtaking, regardless of the type of wine consumed.

The results suggest that a higher sulphite content in wine induces drivers to behave more cautiously under high cognitive load, such as when driving on urban roads while interacting with pedestrians at zebra crossings. Conversely, in the car-following scenario, drivers tended to drive at higher speeds and closer to the vehicle ahead, suggesting different behaviour.

The reasons for this result are not fully explained and warrant further investigation. It is therefore recommended that new research be conducted to determine the effects of sulphites in wines over a wider range of values. As sulphites may have very different effects on individuals, it cannot be excluded that, at certain levels, they may affect driving performance and, more generally, road safety, as the results of this study suggest.

In this study, participants were given wine to keep their BAC within the legal limit. This aspect is crucial and should be clearly stated as it helps in the interpretation of the results. The fact that we did not observe many significant effects suggests that the current legal limits in Italy are effective in maintaining safe driving performance. Furthermore, it is important to emphasise that our study aimed to investigate the effects of sulphites on driving performance, strictly within the limits of legal BAC values and legal restrictions on sulphite content in wines, and not at higher or uncontrolled levels. Therefore, the study’s results should be interpreted in the context of legal conditions rather than as representing the effects of sulphites on a broader or unrestricted scale.

## Supporting information

S1 Data Demographic, physiological responses, and driving performance and safety risk data for each participant. The format is 32 columns by 96 rows. Column 1: (Test_Driver_ID) Identification number of the test driver; Column 2: (Gender) Gender of the test driver (M, F); Column 3: (Age) Age of the test drivers (years); Column 4: (Weight) Weight of the test driver (kg); Column 5: (Height) Height of the test driver (m); Column 6: (BMI) Body Mass Index of the test driver (kg/m^2^); Column 7: (Driving_experience, years) Driving experience of the test driver (years of driving license); Column 8: (Driving_experience, km/year) Driving experience of the test driver (km travelled per year); Column 9: (Sulphite_content) Sulphites content (mg/l, 0 = baseline, 86 = low content, 126 = high content); Column 10: (BAC_F) Final Blood Alcohol Concentration of the test driver after driving (‰); Column 11: (rural_tangent_S) Mean speed along the straight segments in the rural environment (km/h); Column 12: (rural_tangent_SDS) Standard deviation of speed along the straight segments in the rural environment (km/h); Column 13: (rural_tangent_SDLP) Standard deviation of lateral position along the straight segments in the rural environment (m); Column 14; (rural_curve_S) Mean speed along curves in the rural environment (km/h); Column 15: (rural_curve_LP) Lateral position along curves in the rural environment (m); Column 16: (rural_curve_SDLP) Standard deviation of lateral position along curves in the rural environment (m); Column 17: (rural_overtake) Overtaking event in the rural environment (YES, NO); Column 18: (trans_time) Transition time (time needed to adapt the vehicle speed from the extra-urban environment to the urban speed limit, s); Column 19: (trans_length) Transition length (distance needed to adapt the vehicle speed from the extra‑urban environment to the urban speed limit, m); Column 20: (trans_SDS) Standard deviation of speed in the transition zone (km/h); Column 21: (trans_Smax) Maximum speed in the transition zone (km/h); Column 22: (urb_MTTC_dx) Minimum Time-To-Collision (MTTC) in the urban environment when the pedestrian crosses from the right side (s); Column 23: (urb_PET_dx) Post Enchroachment Time (PET) in the urban environment when the pedestrian crosses from the right side (s); Column 24: (urb_overtake) Overtaking event in the urban environment (YES, NO); Column 25: (urb_CF_S) Mean speed during the car-following in the urban environment (km/h); Column 26: (urb_CF_SDS) Standard deviation of speed during the car-following in the urban environment (km/h); Column 27: (urb_CF_Smax) Maximum speed during the car‑following in the urban environment (km/h); Column 28: (urb_CF_LP) Lateral position during the car-following in the urban environment (m); Column 29: (urb_CF_SDLP) Standard deviation of lateral position during the car-following in the urban environment (m); Column 30: (urb_CF_HW) Mean headway during the car-following in the urban environment (s); Column 31: (urb_CF_SDHW) Standard deviation of headway during the car-following in the urban environment (s); Column 32: (urb_CF_minHW) Minimum headway during the car-following in the urban environment (s).
(CSV)

## References

[1] National Highway Traffic Safety Administration. Traffic Safety Facts: 2022 Data. Alcohol-Impaired Driving. DOT HS 813 578. U.S. Department of Transportation. 2024. https://crashstats.nhtsa.dot.gov/Api/Public/ViewPublication/813578

[2] European Transport Safety Council. Progress in reducing drink driving and other alcohol-related road deaths in Europe. 2022. https://etsc.eu/progress-in-reducing-drink-driving-and-other-alcohol-related-road-deaths-in-europe/

[3] Istituto Nazionale di Statistica. Incidenti stradali in Italia—Anno 2023. 2024. https://www.istat.it/wp-content/uploads/2024/07/REPORT-INCIDENTI-STRADALI-2023.pdf

[4] Bassani M, Passalacqua P, Catani L, Bruno G, Spoto A. A driving simulation study on the effects of different wine types on the performance of young drivers. Drug Alcohol Depend. 2021;225:108847. doi: 10.1016/j.drugalcdep.2021.108847 34182375

[5] Fillmore MT, Blackburn JS, Harrison ELR. Acute disinhibiting effects of alcohol as a factor in risky driving behavior. Drug Alcohol Depend. 2008;95(1–2):97–106. doi: 10.1016/j.drugalcdep.2007.12.018 18325693 PMC2376256

[6] Helland A, Jenssen GD, Lervåg L-E, Westin AA, Moen T, Sakshaug K, et al. Comparison of driving simulator performance with real driving after alcohol intake: a randomised, single blind, placebo-controlled, cross-over trial. Accid Anal Prev. 2013;53:9–16. doi: 10.1016/j.aap.2012.12.042 23357031

[7] van Dijken JH, Veldstra JL, van de Loo AJAE, Verster JC, van der Sluiszen NNJJM, Vermeeren A, et al. The influence of alcohol (0.5‰) on the control and manoeuvring level of driving behaviour, finding measures to assess driving impairment: A simulator study. Transportation Research Part F: Traffic Psychology and Behaviour. 2020;73:119–27. doi: 10.1016/j.trf.2020.06.017

[8] Giacosa S, Río Segade S, Cagnasso E, Caudana A, Rolle L, Gerbi V. SO2 in wines: rational use and possible alternatives. Red Wine Technology. Elsevier. 2026. p. 495–510. doi: 10.1016/b978-0-443-33914-1.00025-8

[9] Giacosa S, Parpinello GP, Río Segade S, Ricci A, Paissoni MA, Curioni A, et al. Diversity of Italian red wines: A study by enological parameters, color, and phenolic indices. Food Res Int. 2021;143:110277. doi: 10.1016/j.foodres.2021.110277 33992377

[10] European Commission. Regulation (EC) No 889/2008 of 5 September 2008 amending Regulation (EC) No 834/2007 on organic production and labelling of organic products. Official Journal of the European Union. 2008;1–84. https://eur-lex.europa.eu/legal-content/IT/TXT/PDF/?uri=CELEX:02008R0889-20161107

[11] Suzzi G, Romano P, Zambonelli C. SaccharomycesStrain Selection in Minimizing SO2Requirement During Vinification. Am J Enol Vitic. 1985;36(3):199–202. doi: 10.5344/ajev.1985.36.3.199

[12] Gao YC, Zhang G, Krentz S, Darius S, Power J, Lagarde G. Inhibition of spoilage lactic acid bacteria by lysozyme during wine alcoholic fermentation. Aust J Grape Wine Res. 2002;8(1):76–83. doi: 10.1111/j.1755-0238.2002.tb00214.x

[13] Vally H, Thompson PJ. Role of sulfite additives in wine induced asthma: single dose and cumulative dose studies. Thorax. 2001;56(10):763–9. doi: 10.1136/thorax.56.10.763 11562514 PMC1745927

[14] Ministero delle Infrastrutture e dei Trasporti. Codice della strada (Decreto legislativo n. 285 del 30 aprile 1992 e successive modifiche). Gazzetta Ufficiale della Repubblica Italiana. 1992. https://www.gazzettaufficiale.it/eli/gu/1992/05/18/114/so/74/sg/pdf

[15] Kalinowski A, Humphreys K. Governmental standard drink definitions and low-risk alcohol consumption guidelines in 37 countries. Addiction. 2016;111(7):1293–8. doi: 10.1111/add.13341 27073140

[16] Bassani M, Catani L, Ignazzi A, Piras M. Validation of a fixed-base driving simulator to assess behavioural effects of road geometrics. In: Proceedings of the DSC2018 Europe. 2018;101–8.

[17] Catani L, Bassani M. Anticipatory distance, curvature, and curvature change rate in compound curve negotiation: a comparison between real and simulated driving. Washington, DC; 2018.

[18] Karimi A, Bassani M, Boroujerdian AM, Catani L. Investigation into passing behavior at passing zones to validate and extend the use of driving simulators in two-lane roads safety analysis. Accid Anal Prev. 2020;139:105487. doi: 10.1016/j.aap.2020.105487 32135336

[19] Lioi A, Portera A, Hazoor A, Tefa L, Karimi A, Bassani M. Driver behaviour assessment due to changes in the geometric layout to integrate lateral jet fans in long road tunnels. Transportation Res Interdisciplinary Perspectives. 2024;26:101137. doi: 10.1016/j.trip.2024.101137

[20] Levitt MD, Li R, DeMaster EG, Elson M, Furne J, Levitt DG. Use of measurements of ethanol absorption from stomach and intestine to assess human ethanol metabolism. Am J Physiol. 1997;273(4):G951-7. doi: 10.1152/ajpgi.1997.273.4.G951 9357840

[21] Ministero delle Infrastrutture e dei Trasporti. Norme funzionali e geometriche per la costruzione delle strade. 2001. https://www.mit.gov.it/normativa/decreto-ministeriale-protocollo-6792-del-05112001

[22] Hussain Q, Alhajyaseen WKM, Pirdavani A, Reinolsmann N, Brijs K, Brijs T. Speed perception and actual speed in a driving simulator and real-world: A validation study. Transportation Research Part F: Traffic Psychology and Behaviour. 2019;62:637–50. doi: 10.1016/j.trf.2019.02.019

[23] Bella F. Driving simulator for speed research on two-lane rural roads. Accid Anal Prev. 2008;40(3):1078–87. doi: 10.1016/j.aap.2007.10.015 18460376

[24] Verster JC, Roth T. Standard operation procedures for conducting the on-the-road driving test, and measurement of the standard deviation of lateral position (SDLP). Int J Gen Med. 2011;4:359–71. doi: 10.2147/IJGM.S19639 21625472 PMC3100218

[25] Angioi F, Bassani M. The implications of situation and route familiarity for driver-pedestrian interaction at uncontrolled mid-block crosswalks. Transportation Research Part F: Traffic Psychology and Behaviour. 2022;90:287–99. doi: 10.1016/j.trf.2022.09.003

[26] Tarko AP. Surrogate measures of safety. Safe mobility: challenges, methodology and solutions. Emerald Publishing Limited. 2018. p. 383–405. doi: 10.1108/S2044-994120180000011019

[27] Arun A, Haque MM, Bhaskar A, Washington S, Sayed T. A systematic mapping review of surrogate safety assessment using traffic conflict techniques. Accid Anal Prev. 2021;153:106016. doi: 10.1016/j.aap.2021.106016 33582529

[28] Berthelon C, Gineyt G. Effects of alcohol on automated and controlled driving performances. Psychopharmacology (Berl). 2014;231(10):2087–95. doi: 10.1007/s00213-013-3352-x 24292385

[29] Akaike H. A new look at the statistical model identification. IEEE Trans Automat Contr. 1974;19(6):716–23. doi: 10.1109/tac.1974.1100705

[30] Stone M. Comments on Model Selection Criteria of Akaike and Schwarz. J Royal Statistical Society Series B: Statistical Methodology. 1979;41(2):276–8. doi: 10.1111/j.2517-6161.1979.tb01084.x

[31] Bates D, Mächler M, Bolker B, Walker S. Fitting Linear Mixed-Effects Models Using lme4. J Stat Soft. 2015;67(1). doi: 10.18637/jss.v067.i01

[32] Core Team R. R: A Language and Environment for Statistical Computing. Vienna, Austria: R Foundation for Statistical Computing. 2023.

[33] Lenth R, Singmann H, Love J, Buerkner P, Herve M. Estimated Marginal Means, aka Least-Squares Means. 2021.

[34] Villanueva RAM, Chen ZJ. ggplot2: elegant graphics for data analysis. 2019. doi: 10.1080/15366367.2019.1565254

[35] Baraona E, Abittan CS, Dohmen K, Moretti M, Pozzato G, Chayes ZW, et al. Gender differences in pharmacokinetics of alcohol. Alcohol Clin Exp Res. 2001;25(4):502–7. doi: 10.1097/00000374-200104000-00004 11329488

[36] Kwo PY, Ramchandani VA, O’Connor S, Amann D, Carr LG, Sandrasegaran K, et al. Gender differences in alcohol metabolism: relationship to liver volume and effect of adjusting for body mass. Gastroenterology. 1998;115(6):1552–7. doi: 10.1016/s0016-5085(98)70035-6 9834284

[37] Verster JC, Bervoets AC, de Klerk S, Vreman RA, Olivier B, Roth T, et al. Effects of alcohol hangover on simulated highway driving performance. Psychopharmacology (Berl). 2014;231(15):2999–3008. doi: 10.1007/s00213-014-3474-9 24563184

[38] Howat P, Sleet D, Smith I. Alcohol and driving: is the 0.05% blood alcohol concentration limit justified?. Drug Alcohol Rev. 1991;10(2):151–66. doi: 10.1080/09595239100185211 16840263

[39] Van Dyke NA, Fillmore MT. Laboratory analysis of risky driving at 0.05% and 0.08% blood alcohol concentration. Drug and Alcohol Dependence. 2017;175:127–32. doi: 10.1016/j.drugalcdep.2017.02.005 28412303 PMC5467693

[40] Fuller R. Towards a general theory of driver behaviour. Accid Anal Prev. 2005;37(3):461–72. doi: 10.1016/j.aap.2004.11.003 15784200

[41] Ahlström C, Zemblys R, Finér S, Kircher K. Alcohol impairs driver attention and prevents compensatory strategies. Accid Anal Prev. 2023;184:107010. doi: 10.1016/j.aap.2023.107010 36806077

[42] Underwood G, Ngai A, Underwood J. Driving experience and situation awareness in hazard detection. Safety Science. 2013;56:29–35. doi: 10.1016/j.ssci.2012.05.025

[43] de Craen S, Twisk DAM, Hagenzieker MP, Elffers H, Brookhuis KA. The development of a method to measure speed adaptation to traffic complexity: identifying novice, unsafe, and overconfident drivers. Accid Anal Prev. 2008;40(4):1524–30. doi: 10.1016/j.aap.2008.03.018 18606286

